# Eye care utilization by older adults in low, middle, and high income countries

**DOI:** 10.1186/1471-2415-12-5

**Published:** 2012-04-03

**Authors:** Claudia Vela, Elodie Samson, Maria Victoria Zunzunegui, Slim Haddad, Marie-Josée Aubin, Ellen E Freeman

**Affiliations:** 1 Hôpital Maisonneuve-Rosemont Recherche ophtalmologie, F131 5415 boulevard de l'Assomption, Montreal, QC H1T 2M4, Canada; 2 Centre de Recherche de Centre Hospitalier de l'Université de Montréal, Montreal, Quebec, Canada; 3 Department of Ophthalmology, Université de Montréal, Montreal, Quebec, Canada

## Abstract

**Background:**

The risk of visual impairment increases dramatically with age and therefore older adults should have their eyes examined at least every 1 to 2 years. Using a world-wide, population-based dataset, we sought to determine the frequency that older people had their eyes examined. We also examined factors associated with having a recent eye exam.

**Methods:**

The World Health Surveys were conducted in 70 countries throughout the world in 2002-2003 using a random, multi-stage, stratified, cluster sampling design. Participants 60 years and older from 52 countries (n = 35,839) were asked "When was the last time you had your eyes examined by a medical professional?". The income status of countries was estimated using gross national income per capita data from 2003 from the World Bank website. Prevalence estimates were adjusted to account for the complex sample design.

**Results:**

Overall, only 18% (95% CI 17, 19) of older adults had an eye exam in the last year. The rate of an eye exam in the last year in low, lower middle, upper middle, and high income countries was 10%, 24%, 22%, and 37% respectively. Factors associated with having an eye exam in the last year included older age, female gender, more education, urban residence, greater wealth, worse self-reported health, having diabetes, and wearing glasses or contact lenses (p < 0.05).

**Conclusions:**

Given that older adults often suffer from age-related but treatable conditions, they should be seen on a regular basis to prevent visual impairment and its disabling consequences.

## Background

Older adults have the highest rates of eye disease and visual impairment. Ophthalmic and optometric organizations recommend that older adults visit an eye care professional every 1-2 years to have a comprehensive eye exam [1,2]. Those with diabetes should visit an eye care professional every year [1,2]. Several studies have examined compliance with these recommendations in high-income countries like the United States, Australia, and Canada [3-6]. For example, in the United States, 65-69% of adults aged 50 years and older visited an eye care provider in the last year [4]. In Australia, 62% of older Australians visited an eye care professional in the last 2 years [5]. However, very few studies examining this issue have been done in low and middle income countries with the exception of China, India, and Iran [7-9]. In rural India, 64% of adults ages 40 years and older had never had an eye exam. We sought to determine the prevalence of an eye exam in the last year and its associated factors in 52 high, middle, and low income countries throughout the world using data from the World Health Survey (WHS).

## Methods

### World health survey

#### Study Population

The WHS was coordinated by the World Health Organization (WHO) to collect population-based, nationally representative, high quality cross-sectional data from 70 countries within 6 world regions [10]. This paper focuses on the 52 countries that asked about eye care utilization including 18 African countries, 13 European countries, 7 countries from Central and South America, 4 Middle Eastern countries, 5 from Southeast Asia, and 5 from the Western Pacific in 2002-2003. Survey institutions were selected by WHO in each country and these institutions carried out the survey according to WHS procedures. Informed consent was obtained from all participants and ethics approval was obtained from every institution that was involved in the study.

#### Sampling Strategy

A multi-stage stratified random cluster sampling strategy was used to identify the participants to be contacted in each country. All sampling plans were reviewed by WHO before implementation. Strata were created based on 3 factors: region, socioeconomic status, and presence of a healthcare facility. Lists of households were obtained from population registries, voter lists, manual enumeration, or other methods. Households within the sampling units were randomly selected from these lists. Each member of the household was listed by the household informant. Within each household, an adult 18 years or older was randomly selected using a Kish table to complete the survey. Non-response was carefully documented. Response rates were very good with an average household response rate of 87% and an average individual response rate of 97%.

#### Survey

All surveys were interviewer-administered in person in local languages. Questionnaires were translated into 68 local languages using standard techniques. Briefly, forward translation was done locally by a bilingual multidisciplinary group. Back-translation was then done by an independent group. A review of the back-translation was also done at WHO. Any discrepancies were resolved. A review of the translated instrument was then done by a panel of experts. Detailed background information was obtained on the age, gender, urban versus rural dwelling, and education of the participant. Participants were asked to rate their general health status and whether they wore eyeglasses or contact lenses. Participants who were aged 60 years and older were asked "When was the last time you had your eyes examined by a medical professional?".

The wealth of the participant was estimated by measuring asset ownership using the widely used method of Filmer and Pritchett [11]. This asset index had good internal coherence and robustness to the selection of variables used [11]. This measure is considered to represent the household's long-run economic status rather than its current economic status. WHS participants were asked about their ownership of various items (i.e. bicycle, television, computer).

#### Income Status of Country

We also obtained data on the income status of the 52 countries from the World Bank website using data from 2003 [12]. According to the website, gross national income was converted to U.S. dollars using the Atlas method and was divided by the mid-year population. The Atlas method of conversion is used by the World Bank to smooth fluctuations in prices and exchange rates. We categorized this variable into 4 categories using World Bank classifications. The 4 categories were low (<$766), lower middle ($766-$3035), upper middle ($3036-$9385), and high income (> $9385) [12].

### Data analysis

There were 46,209 people who reported their age to be 60 years or older. We limited our analyses to the 35,839 respondents from the 52 countries who answered the question on the time of their last eye exam who reported their age to be 60 years or older (78% of the total). Means, standard deviations, and percentages were estimated. Prevalence rates were adjusted for the complex survey design although 3 countries (Slovenia, Guatamala, Zambia) did not report survey design information and therefore unweighted estimates are given for these 3 countries. Logistic regression analyses were performed to identify factors associated with having an eye exam in the last year. A random effects model with an exchangeable correlation structure was used to account for the multi-level nature of the data (people within countries). Principal components analysis was used to determine the weights for the index of the asset variables according to the method of Filmer and Pritchett [11]. The index was divided into tertiles for use in the regression model. Stata Version 11 was used for the analyses (StataCorp, College Station Texas, USA) while the map was created in SAS Version 9.2 (Cary, North Carolina, USA).

## Results

The study population is described in Table 1. Respondents come from 52 countries with the average sample size of participants aged 60 years and older being 692 people per country with a range of 46 (United Arab Emirates) to 6298 (Mexico) people. The majority of the participants come from low or lower middle income countries (65%). Correspondingly, 49% of the participants had less than a primary school education.

**Table 1:** Descriptive statistics of WHS study population who are 60 years and older

Variable	Overall Mean (SD) or % N = 35,839
Income status of country of origin	
Low	31%
Lower middle	34%
Upper middle	28%
High	7%

Age, Years	70 (8)

Female gender	56%

Highest education level	
No formal schooling	33%
Less than primary school	16%
Completed primary school	20%
Completed secondary school	19%
Completed high school	7%
Completed college/university	5%
Completed post-graduate degree	1%

Setting	
Urban or Semi-Urban	51%
Rural	49%

Health status	
Very good	8%
Good	28%
Fair	42%
Bad or very bad	22%

Diabetes	9%

**Figure 1:**
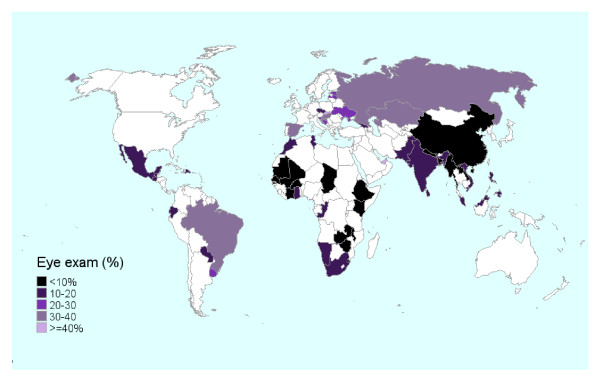
**Map of WHS countries showing the percentage of participants aged 60 years and older who reported an eye exam in the last year**.

Overall, only 18% (95% CI 17, 19) had an eye exam in the last year (Table 2). The percentage of people having an eye exam in the last year in low, lower middle, upper middle, and high income countries was 10%, 24%, 22%, and 37% respectively (see list of countries in footnote to Table 2). A map showing the percentages by country can be found in Figure 1. The countries in the darkest colors have the lowest rates of an eye exam in the last year with countries in Africa and Southeast Asia having the lowest rates. Strikingly, 38% of people overall reported never having an eye exam (Table 2). The rate of never having an eye exam in low, lower middle, upper middle, and high income countries was 61%, 20%, 26%, and 5% respectively. Of those reporting a diagnosis of diabetes (9%), 32% reported an eye exam in the last year while 19% had never had an exam.

**Table 2:** Ocular or eye exam characteristics by income status of the country of the participant

Variable	Overall %* N = 35,839	LIC %* N = 11,020	LMIC %* N = 12,157	UMIC %* N = 10,057	HIC %* N = 2,605
Wears glasses or contacts	47%	27%	62%	64%	83%

When was your last eye exam					
Within last 12 months	18%	10%	24%	22%	37%
1-2 years ago	16%	9%	21%	20%	27%
3-4 years ago	9%	5%	11%	12%	13%
5 years ago	3%	3%	4%	3%	3%
More than 5 years ago	16%	12%	20%	17%	15%
Never	38%	61%	20%	26%	5%

Diabetics					
Eye exam in last 12 months	32%	18%	37%	36%	43%
Never had an eye exam	19%	38%	11%	14%	2%

LIC = low income countries (Bangladesh, Burkina Faso, Chad, Comoros, Congo, Cote d'Ivoire, Ethiopia, Ghana, India, Kenya, Lao, Malawi, Mali, Mauritania, Myanmar, Nepal, Pakistan, Senegal, Vietnam, Zambia, Zimbabwe)

LMIC = lower middle income countries (Bosnia Herzegovina, Brazil, China, Dominican Republic, Ecuador, Georgia, Guatemala, Kazakhstan, Morocco, Namibia, Paraguay, Philippines, Russian Federation, South Africa, Sri Lanka, Swaziland, Tunisia, Ukraine)

UMIC = upper middle income countries (Croatia, Czech Republic, Estonia, Hungary, Latvia, Malaysia, Mauritius, Mexico, Slovakia, Uruguay)

HIC = high income countries (Slovenia, Spain, United Arab Emirates)

*Data are weighted to account for the survey design except for 3 countries (Guatemala, Zambia, and Slovenia) which did not include survey design data

We examined demographic, health, and ocular factors associated with having an eye exam in the last year (Table 3). Older people, women, those with more education, those with greater wealth, those with worse reported health, those with diabetes, and those who wore glasses or contact lenses were more likely to report having an eye exam in the last year (P < 0.01). Those who lived in a rural area were less likely to report having an eye exam in the last year compared to those living in an urban or semi-urban area (P < 0.01). There was no evidence that the factors differed by the income status of the country as odds ratios were very similar between income strata (data not shown).

**Table 3:** Factors independently associated with having an eye exam in the last year from random effect multiple logistic regression model

n = 31,530	OR	95% CI
Age	1.01	1.00, 1.01

Female gender	1.17	1.10, 1.25

Highest education level		
No formal schooling	1.00	
Less than primary school	1.27	1.12, 1.43
Completed primary school	1.29	1.14, 1.45
Completed secondary school	1.50	1.31, 1.71
Completed high school	1.99	1.70, 2.33
Completed college/university	2.00	1.68, 2.38
Completed post-graduate degree	2.84	2.11, 3.82

Wealth index of participant		
Lowest category	1.00	
Middle category	1.28	1.19, 1.38
Upper category	1.62	1.46, 1.80

Setting		
Urban	1.00	
Rural	0.75	0.70. 0.81

Health status		
Very good	1.00	
Good	1.17	1.01, 1.35
Fair	1.38	1.20, 1.59
Bad or very bad	1.58	1.36, 1.84

Diabetes	1.67	1.53, 1.83

Wears glasses or contact lenses	2.26	2.10, 2.45

## Discussion

Older adults have the highest rates of visual impairment due to age-related eye diseases and conditions like cataract, glaucoma, presbyopia, and age-related macular degeneration [13,14]. Also, older patients with diabetes are at risk of developing diabetic retinopathy [15]. Therefore, older adults, more than any other age group, need to have routine eye examinations at least every 1-2 years. Most older people who already know that they have a chronic eye disease need to be seen at least every year, if not more frequently.

To our knowledge, this is the first study to examine eye care utilization on a world-wide basis. Very few studies have examined the rate of eye care utilization in low or middle income countries [7-9]. We found very low rates of eye care utilization in the world with an overall rate of only 18% of older adults having seen an eye care professional in the last year. The rate varied 4-fold depending on the income status of the country.

Prior research on eye care utilization has typically focused on high income countries like the United States, Australia, and Canada [3-6,16-18]. Three studies from these high-income countries reported that 60-70% of older adults visited an eye care provider in the last year [3-5]. In the WHS data, only 3 of the 20 high-income countries asked the question on eye exams (Slovenia, Spain, and the United Arab Emirates (UAE)). The rates of an eye exam in the last year in those aged 60 years and older from these three countries were 27% (unweighted), 37%, and 47% respectively. These rates are much lower than the rates seen in the United States, Australia, and Canada.

In low or middle income countries, we only found three studies that reported on this topic. In a population-based study in Tehran, Iran, an upper middle income country, 13% of adults aged 60 years and older had never seen an eye care professional [7]. Iran did not participate in the WHS so we are unable to compare our results. In a study of people with diabetes in China, a lower middle income country, 43% of urban people and 69% of rural people had never had an eye exam [9]. There were not enough people who reported having diabetes living in rural China in the WHS to examine this. However, 46% of urban and 85% of rural Chinese WHS participants reported never having an eye exam. In a study done in rural India, a low income country, 64% of people aged 40 years and older had never had an eye exam [8]. In the WHS data from rural India, we found that 55% of adults aged 60 and older reported never having an eye exam. Because the same age ranges were not included, it is difficult to directly compare the results.

Certain factors were independently related to eye care utilization regardless of the income status of the country. Men had lower rates of eye exams than women. This finding is consistent with other literature [5,6,17,19]. Also, those with lower education had lower rates of eye exams. This finding is also consistent with other literature [16,19,20]. If people do not understand that vision loss is not a normal part of aging and that services may exist to correct this vision loss, they are not likely to have an eye exam. It is also possible that education is confounded with other factors that affect eye exam utilization such as income, culture, or empowerment. Independent from education, those in rural areas were less likely to have an eye exam in the last year than those in urban areas. This may be due to the difficulty in finding an eye care provider in rural areas. Other studies have also documented a lower rate of eye care utilization in rural versus urban areas [9,21]. Those who were in worse self-reported health were more likely to have had an eye exam, which suggests that contact with a healthcare provider may have spurred an eye exam. Those who had diabetes were more likely to have had an eye exam in the last year although only 32% had had an eye care exam in the last year. Current recommendations are that diabetics should see an eye care professional every year. Those who wore glasses or contact lenses were more likely to see an eye care professional in the last year, which is important since refractive status can change and needs to be evaluated on a regular basis.

Those with higher income in the WHS were more likely to have an eye exam in the last year. Few prior studies have examined income. Those that did report inconsistent findings on this relationship. In China, monthly income was not associated with use of eye care services in diabetic patients [9]. In high income countries like the United States, women with a higher income were more likely to have had an eye exam in the last 2 years [20]. Also in the U.S., using data from the National Health Interview Survey, the probability of having a dilated eye exam in those with an eye disease increased with higher income status [17].

Our findings on eye care utilization are consistent with other literature showing that gender, education, and rural residence are related to health services utilization. Men are reported to use less health care services than women including general practice utilization, hypertension and diabetes treatment [22-25] although men may be more likely to be hospitalized [26,27]. People with less education are less likely to use preventive health services (mammogram, Pap test, dentist) [28,29], maternal health care [30], and reproductive services [31]. One study found that health literacy significantly mediated educational disparities in the receipt of the influenza vaccine in older adults [32]. People living in rural areas in a variety of countries are reported to use less healthcare services including antenatal care [33,34], dental services [35,36], physician visits [37], and immunizations [38] compared to people living in urban or suburban areas.

The WHS is a rich, under-utilized source of population-based data from around the world. We augmented the WHS data with data from the World Bank on the income status of the country. Most of the countries had very good response rates which allows us to calculate prevalence estimates that are representative of the entire country. Limitations of the WHS data include that we do not have comprehensive data on barriers to eye care utilization or presence of an eye disease via an eye exam. Eye care utilization is based on self-report which could lead to misclassification if participants do not have a good memory of their last eye exam. We do not know whether the participant visited an ophthalmologist/optometrist/or other medical professional. Only 78% of those aged 60 years and older answered the question on eye care utilization although 75% of this missing data came from upper middle or high income countries which simply did not administer this question. The rate of missing data for this question in low and lower middle income countries was 10% and 10% respectively. The study design is cross-sectional which prohibits us from examining trends in eye care utilization over time and from examining the temporality of the exposures and the outcome.

## Conclusions

These global data indicate that eye care utilization in older adults in much of the world is terribly low. Consequences of vision loss in older adults go beyond the inability to see since vision loss is a determinant of disability and dependency in old age [39,40]. Given that older adults often suffer from age-related but treatable conditions such as cataract, presbyopia, glaucoma, and age-related macular degeneration, they should be seen on a regular basis to minimize the risk of visual impairment and blindness.

## Competing interests

The authors declare that they have no competing interests.

## Authors' contributions

CV helped with data analysis and the literature review. ES constructed the World Health Survey world-wide dataset and cleaned the data. EF supervised CV and ES and performed some of the data analysis. All authors (CV, ES, MVZ, SH, MJA, and EF) provided input on the interpretation of the results and the manuscript and gave final approval of the manuscript.

## Pre-publication history

The pre-publication history for this paper can be accessed here:

http://www.biomedcentral.com/1471-2415/12/5/prepub
